# Susceptibility *in vitro* of canine methicillin-resistant and -susceptible staphylococcal isolates to fusidic acid, chlorhexidine and miconazole: opportunities for topical therapy of canine superficial pyoderma

**DOI:** 10.1093/jac/dkv056

**Published:** 2015-03-05

**Authors:** S. M. Clark, A. Loeffler, R. Bond

**Affiliations:** Department of Clinical Sciences and Services, Royal Veterinary College, Hawkshead Lane, North Mymms, Hatfield, Hertfordshire AL9 7TA, UK

**Keywords:** MRSA, *Staphylococcus pseudintermedius*, dogs, synergy

## Abstract

**Objectives:**

Increasing multidrug resistance amongst canine pathogenic staphylococci has renewed interest in topical antibacterial therapy for skin infections in the context of responsible veterinary prescribing. We therefore determined the activity *in vitro* of three clinically relevant topical agents and synergism between two of them against *Staphylococcus pseudintermedius* and *Staphylococcus aureus*.

**Methods:**

The MICs of fusidic acid (*n* = 199), chlorhexidine (*n* = 198), miconazole (*n* = 198) and a 1:1 combination of miconazole/chlorhexidine (*n* = 198) were determined for canine isolates [50 MRSA and 49 methicillin-resistant *S. pseudintermedius* (MRSP), 50 MSSA and 50 methicillin-susceptible *S. pseudintermedius* (MSSP)] collected from the UK and Germany using an agar dilution method (CLSI VET01-A4). Fractional inhibitory concentration (FIC) indices were calculated to assess the interaction of miconazole with chlorhexidine.

**Results:**

MICs of each drug/combination were significantly (*P* < 0.0005) higher for *S. aureus* when compared with *S. pseudintermedius*. Most strains (*n* = 172) had an MIC of fusidic acid of ≤0.03 mg/L (MIC ≥64 mg/L, *n* = 5 MRSA). All strains had MICs of chlorhexidine of 0.5–4 mg/L, except for one MRSA (MIC = 8 mg/L). All but four strains had MICs of miconazole of 1–4 mg/L (MIC = 16 mg/L, *n* = 3; MIC = 256 mg/L, *n* = 1). Miconazole/chlorhexidine (1:1 ratio) had a synergistic effect against 49/50 MRSA, 31/50 MSSA, 12/49 MRSP and 23/49 MSSP.

**Conclusions:**

Since the majority of these staphylococci, including methicillin-resistant isolates, had MICs that should be readily exceeded by topical skin application of these agents, their therapeutic efficacy for canine superficial pyoderma should be assessed. The synergistic interaction shown *in vitro* supports further clinical evaluation of miconazole/chlorhexidine combination therapy for staphylococcal infection.

## Introduction

The alarming increase in canine skin infections caused by methicillin-resistant *Staphylococcus pseudintermedius* (MRSP) and MRSA^1^ paralleled by recognition of zoonotic (and reverse-zoonotic) infections,^2^ highlights the urgent need to develop strategies to limit further emergence of MDR strains. Topical antibacterial therapy can provide an alternative treatment option for many dogs with bacterial skin infections and thus limit the need for oral antibiotics.^3^ Licensed drugs include fusidic acid, chlorhexidine and a 1:1 shampoo combination of miconazole and chlorhexidine.

Although there is concern in human medicine over reduced efficacy of agents such as chlorhexidine and fusidic acid,^4,5^ clinical evidence for treatment failure remains inconsistent. Assessment of resistance is hampered by lack of breakpoint standards for agents used topically. In dog-derived staphylococci, MICs of fusidic acid, chlorhexidine and miconazole have been low,^6–8^ but geographical differences can be expected.^9,10^ Since previous MIC studies have evaluated mainly North American staphylococcal isolates,^7,8^ we determined current susceptibility *in vitro* of dog-derived European MRSA and MRSP strains and their methicillin-susceptible counterparts to fusidic acid, chlorhexidine and miconazole, and investigated the potential for synergistic interaction between miconazole and chlorhexidine.

## Materials and methods

### Bacterial isolates

Coagulase-positive staphylococci [50 MRSA and 50 MSSA obtained in 2005–07 and 49 MRSP and 50 methicillin-susceptible *S. pseudintermedius* (MSSP) obtained in 2010–13] isolated from dogs were randomly selected from our collection. *Staphylococcus aureus* isolates were from canine infections in a UK-wide study.^11^ Examples of *S. pseudintermedius* comprised clinical isolates (Germany, 25 MRSP and 25 MSSP; UK, 24 MRSP) and carriage isolates (UK, 25 MSSP). Species identification and methicillin resistance were confirmed using both phenotypic and genotypic (*nuc*, *mec*A) methods.^12,13^

### MIC determination

MICs were determined by agar dilution (CLSI VET01-A4).^14^ Prior to MIC determination, strains were subcultured twice on blood agar base (CM0271, Oxoid) containing 5% sheep blood (TCS Biosciences, Buckingham, UK) at 35°C for 24 h. Stock solutions were prepared at 10× final concentration in distilled water (fusidic acid sodium salt F0881, chlorhexidine digluconate C9394; Sigma-Aldrich Inc.) or 1% DMSO (miconazole nitrate PHR1163; Sigma), adjusted for drug potency.^14^ Final concentrations of the active fraction ranged from 0.015 to 2048 mg/L for fusidic acid and from 0.03 to 256 mg/L for chlorhexidine, miconazole and a 1:1 combination of chlorhexidine and miconazole.^14^ Discrepancy between duplicate MICs was accepted provided they varied by only one dilution; in such cases, the higher value was identified as the final MIC. *S. aureus* ATCC 25923, *S. aureus* ATCC 29663 and *S. pseudintermedius* LMG 22219 were included for quality control purposes. Three MRSA strains previously reported with low, medium and high MICs of fusidic acid^6^ were also included for comparative purposes.

### Fractional inhibitory concentration (FIC) indices

The FIC index was calculated to analyse drug interaction of chlorhexidine and miconazole when used in the 1:1 combination, using the formula ΣFIC = FIC_chlorhexidine_ + FIC_miconazole_ = (MIC_CM_/MIC_C_) + (MIC_CM_/MIC_M_), where MIC_C_ and MIC_M_ are the MICs of chlorhexidine and miconazole alone, respectively, and MIC_CM_ is the MIC of the two drugs in combination. An FIC index of ≤0.5 represented synergy, an FIC index of >4 represented antagonism and an FIC index of >0.5–4 represented no interaction.^15^

### Statistical analysis

SPSS version 21 (IBM UK Ltd, Portsmouth, UK) was used, with *P* < 0.05 for significance. Since data were not normally distributed (Shapiro–Wilk test), the MIC values for different bacterial groups were compared using the Kruskal–Wallis test. *Post hoc* comparisons were performed using Mann–Whitney *U*-tests with Holm–Bonferroni adjustments.

## Results

One MSSP strain failed to grow in studies of MICs of chlorhexidine, miconazole and miconazole plus chlorhexidine. MICs varied by a single dilution between replicates on only five occasions.

### Fusidic acid

The MICs of fusidic acid amongst the field collection ranged from ≤0.015 to 384 mg/L (median ≤0.015 mg/L); 172 of 199 isolates had MICs ≤0.03 mg/L and 192 isolates had MICs ≤4 mg/L (Table 1). Seven isolates had higher MICs of 16–384 mg/L, comprising five MRSA (384 mg/L, *n* = 4; 64 mg/L, *n* = 1) and single examples of MSSA and MRSP (both 16 mg/L). Overall, MICs were higher (*P* < 0.0005) for *S. aureus* isolates (median 0.03 mg/L, *n* = 100) than *S. pseudintermedius* isolates (median ≤0.015 mg/L, *n* = 99), though there was no significant difference in MICs between methicillin-susceptible and resistant isolates within each species.

**Table 1.** MICs of fusidic acid for 199 coagulase-positive staphylococcal isolates from dogs and MICs of chlorhexidine, miconazole and a 1:1 combination of miconazole/chlorhexidine for 198 coagulase-positive staphylococcal isolates from dogs

Drug	Bacterial type	MIC (mg/L)																Median (IQR)	MIC_50_	MIC_90_
		≤0.015	(≤)0.03	0.06	0.125	0.25	0.5	1	2	4	8	16	32	64	128	256	384			
Fusidic acid	MRSA	8	34	0	0	3	0	0	0	0	0	0	0	1	0	0	4	0.03 (0.03–0.03)	0.03	0.25
	MSSA	24	13	0	0	1	1	6	2	2	0	1	0	0	0	0	0	0.03 (≤0.015–0.3125)	0.03	1
	MRSP	46	1	0	0	0	0	1	0	0	0	1	0	0	0	0	0	≤0.015 (≤0.015 to ≤0.015)	≤0.015	≤0.015
	MSSP	41	5	0	0	0	0	0	1	3	0	0	0	0	0	0	0	≤0.015 (≤0.015 to ≤0.015)	≤0.015	0.03
Chlorhexidine	MRSA	not tested	0	0	0	0	0	0	0	49	1	0	0	0	0	0	0	4 (4–4)	4	4
	MSSA	not tested	0	0	0	0	1	22	22	5	0	0	0	0	0	0	0	2 (1–2)	2	2
	MRSP	not tested	0	0	0	0	19	10	18	2	0	0	0	0	0	0	0	1 (0.5–2)	1	2
	MSSP	not tested	0	0	0	0	21	25	0	3	0	0	0	0	0	0	0	1 (0.5–1)	1	1
Miconazole	MRSA	not tested	0	0	0	0	0	0	43	6	0	1	0	0	0	0	0	2 (2–2)	2	4
	MSSA	not tested	0	0	0	0	0	4	32	12	0	1	0	0	0	1	0	2 (2–4)	2	4
	MRSP	not tested	0	0	0	0	0	22	27	0	0	0	0	0	0	0	0	2 (1–2)	2	2
	MSSP	not tested	0	0	0	0	0	43	4	1	0	1	0	0	0	0	0	1 (1–1)	1	2
Miconazole plus chlorhexidine (1:1)	MRSA	not tested	0	0	0	0	45	4	1	0	0	0	0	0	0	0	0	0.5 (0.5–0.5)	0.5	0.5
	MSSA	not tested	0	0	0	7	41	0	1	0	1	0	0	0	0	0	0	0.5 (0.5–0.5)	0.5	0.5
	MRSP	not tested	0	0	0	2	47	0	0	0	0	0	0	0	0	0	0	0.5 (0.5–0.5)	0.5	0.5
	MSSP	not tested	0	0	0	40	8	0	1	0	0	0	0	0	0	0	0	0.25 (0.25–0.25)	0.25	0.5

MRSA, *n* = 50; MSSA, *n* = 50; MRSP, *n* = 49; and MSSP, *n* = 50 for fusidic acid and *n* = 49 for chlorhexidine, miconazole and miconazole plus chlorhexidine.

MICs for reference isolates were low (ATCC 25932, 0.03 mg/L; ATCC 29663 and LMG 22219, ≤0.015 mg/L), as expected. The isolates included for internal comparative purposes had MICs comparable to previous determinations [A004, 0.12 (previous) and 0.03 (present) mg/L; A013, 2 and 1 mg/L; and A016, >128 and 384 mg/L].

### Chlorhexidine

The MICs of chlorhexidine ranged from 0.5 to 8 mg/L (median 2 mg/L; Table 1). The MICs for MRSA (*n* = 50) were remarkably uniform, with 49 out of 50 isolates having an MIC of 4 mg/L; these values were higher (*P* < 0.0005) than those for all other bacterial groups. Overall, the *S. aureus* isolates (median 4 mg/L, *n* = 100) had higher MICs than the *S. pseudintermedius* isolates (median 1 mg/L, *n* = 98) (*P* < 0.0005). The MICs did not vary significantly between MRSP (*n* = 49) and MSSP (*n* = 49) (*P* = 0.055).

### Miconazole

The MICs of miconazole ranged from 1 to 256 mg/L (median 2 mg/L); 175 of 198 isolates had MICs of 1 or 2 mg/L and 19 had MICs of 4 mg/L (Table 1). Four isolates had higher MICs, comprising single examples of MRSA, MSSA and MSSP with MICs of 16 mg/L and one MSSA with an MIC of 256 mg/L. *S. aureus* isolates (median 2 mg/L, *n* = 100) had higher MICs than *S. pseudintermedius* isolates (median 1 mg/L, *n* = 98) (*P* < 0.0005). There was no difference (*P* = 0.415) in MIC between the MRSA and MSSA groups; however, the MRSP group (median 2 mg/L, *n* = 49) had a significantly (*P *< 0.0005) higher MIC than the MSSP group (median 1 mg/L, *n* = 49).

### Miconazole and chlorhexidine in combination

The MICs of the 1:1 combination of miconazole and chlorhexidine ranged from 0.25 to 8 mg/L (median 0.5 mg/L; Table 1); 190 of 198 isolates had an MIC of 0.25 or 0.5 mg/L and all but one MSSA isolate had an MIC of ≤2 mg/L. The MICs for the *S. aureus* isolates (median 0.5 mg/L) exceeded (*P* < 0.0005) those for the *S. pseudintermedius* isolates (median 0.5 mg/L), and the MICs for the MSSP group (median 0.25 mg/L) were lower (*P* < 0.0005) than for the other three groups. The MSSA isolate whose miconazole MIC was 256 mg/L had an MIC of 8 mg/L for the combination.

A synergistic interaction between miconazole and chlorhexidine (FIC index ≤0.5) was observed for 80 out of 100 *S. aureus* isolates and 35 out of 98 *S. pseudintermedius* isolates (Table 2). Antagonistic interactions were not observed; the other strains fell into the ‘no interaction’ group as defined by Odds.^15^ EUCAST guidelines^16^ consider that FIC indices from 0.5 to 1 indicate additivity; by this definition a further 62 strains demonstrated additive effects (MRSA, *n* = 1; MSSA, *n* = 18; MRSP, *n* = 19; MSSP, *n* = 24).

**Table 2.** FIC indices for a 1:1 combination of chlorhexidine and miconazole

Bacterial group	Synergy^15^ (≤0.5)	No interaction^15^			Antagonism^15^ (>4)
		>0.5–1	>1–2	>2–4	
MRSA (*n* = 50)	49	1	0	0	0
MSSA (*n* = 50)	31	18	0	1	0
MRSP (*n* = 49)	12	19	18	0	0
MSSP (*n* = 49)	23	24	2	0	0
Total	115	62	20	1	0

## Discussion

The low MICs of fusidic acid, miconazole and chlorhexidine for the great majority of these canine staphylococcal isolates *in vitro* provide strong support for their use topically in clinical cases of surface and superficial pyoderma, including cases caused by methicillin-resistant bacteria.

The interpretation of MIC values in the context of susceptibility/resistance is hindered by the absence of defined breakpoints; whilst EUCAST guidelines indicate a value of 2 mg/L for fusidic acid when used against *S. aureus*, this is of questionable value for canine skin infections treated topically rather than systemically.

The prevalence of high fusidic acid MICs for human-derived *S. aureus* appears related to geographical variation in prescribing practice in human medicine;^17^ 10.7% of human-derived European *S. aureus* had MICs ≥2 mg/L^9^ compared with only 0.3% of isolates from North America, where fusidic acid usage is infrequent.^10^ The comparable frequency of fusidic acid MICs ≥2 mg/L amongst our European canine MRSA isolates (10.0%) is perhaps not surprising since these strains likely originate from human sources.^6^ The strikingly uniform MICs of chlorhexidine, almost exclusively in the narrow range of 0.5–4 mg/L, are remarkably similar to those reported previously for MRSA isolates from human sources worldwide.^18^ Similarly, the MICs of miconazole in the present study are comparable to those in previous reports indicating good efficacy against staphylococci.^8^

The frequent synergistic activity of the combination of chlorhexidine and miconazole indicates potential clinical value as a combination treatment in dogs, especially as they lack a critical role in human medicine.

Monitoring for the presence, and potential for transfer, of resistance between populations of MRSA on dogs and humans, and between canine *S. pseudintermedius* and human *S. aureus* strains is warranted. Genetic studies are also required to explain the rare higher MICs that were identified in individual isolates. Previous authors have reported maximum values in the order of 16 mg/L miconazole for *S. aureus* and 8 mg/L fusidic acid for *S. pseudintermedius*; the value of 16 mg/L fusidic acid seen for a single MRSP strain might correspond to ‘low-level fusidic acid resistance’^17^ and explanation is needed for the single high MIC of miconazole of 256 mg/L.

This study provides further evidence to support topical therapies as an alternative to systemic antibacterial drugs in superficial pyoderma. Further work is needed to confirm the clinical efficacy of these topical treatments.

## Funding

This study was funded by a Biotechnology and Biological Sciences Research Council (Swindon, UK) industrial CASE scholarship, in partnership with Dechra Veterinary Products Limited (DVP), Shropshire, UK (BB/K011952/1).

## Transparency declarations

The authors' group has previously received funding from DVP in support of laboratory research and clinical teaching.
